# The Impact of Machine Learning Mortality Risk Prediction on Clinician Prognostic Accuracy and Decision Support: A Randomized Vignette Study

**DOI:** 10.1177/0272989X251349489

**Published:** 2025-07-04

**Authors:** Ravi B. Parikh, William J. Ferrell, Anthony Girard, Jenna White, Sophia Fang, Justin E. Bekelman, Marilyn M. Schapira

**Affiliations:** Department of Medicine, Perelman School of Medicine, University of Pennsylvania, Philadelphia, PA, USA; Department of Medical Ethics and Health Policy, Perelman School of Medicine, University of Pennsylvania, Philadelphia, PA, USA; Penn Center for Cancer Care Innovation, Abramson Cancer Center, University of Pennsylvania, Philadelphia, PA, USA; Corporal Michael J. Crescenz VA Medical Center, Philadelphia, PA, USA; Department of Medical Ethics and Health Policy, Perelman School of Medicine, University of Pennsylvania, Philadelphia, PA, USA; Penn Center for Cancer Care Innovation, Abramson Cancer Center, University of Pennsylvania, Philadelphia, PA, USA; Department of Medical Ethics and Health Policy, Perelman School of Medicine, University of Pennsylvania, Philadelphia, PA, USA; Department of Medical Ethics and Health Policy, Perelman School of Medicine, University of Pennsylvania, Philadelphia, PA, USA; Penn Center for Cancer Care Innovation, Abramson Cancer Center, University of Pennsylvania, Philadelphia, PA, USA; Department of Operations Research and Financial Engineering, Princeton University, Princeton, NJ, USA; Department of Medicine, Perelman School of Medicine, University of Pennsylvania, Philadelphia, PA, USA; Department of Medical Ethics and Health Policy, Perelman School of Medicine, University of Pennsylvania, Philadelphia, PA, USA; Penn Center for Cancer Care Innovation, Abramson Cancer Center, University of Pennsylvania, Philadelphia, PA, USA; Department of Medicine, Perelman School of Medicine, University of Pennsylvania, Philadelphia, PA, USA; Penn Center for Cancer Care Innovation, Abramson Cancer Center, University of Pennsylvania, Philadelphia, PA, USA; Corporal Michael J. Crescenz VA Medical Center, Philadelphia, PA, USA

**Keywords:** machine learning, artificial intelligence, prognosis, palliative care, non-small cell lung cancer

## Abstract

**Background:**

Machine learning (ML) algorithms may improve the prognosis for serious illnesses such as cancer, identifying patients who may benefit from earlier palliative care (PC) or advance care planning (ACP). We evaluated the impact of various presentation strategies of a hypothetical ML algorithm on clinician prognostic accuracy and decision making.

**Methods:**

This was a randomized clinical vignette survey study among medical oncologists who treat metastatic non-small-cell lung cancer (mNSCLC). Between March and June 2023, clinicians were shown 3 vignettes of patients presenting with mNSCLC. The vignettes varied by prognostic risk, as defined from the Lung Cancer Prognostic Index (LCPI). Clinicians estimated life expectancy in months and made recommendations about PC and ACP. Clinicians were then shown the same vignette with a hypothetical survival estimate from a black-box ML algorithm; clinicians were randomized to receive the ML prediction using absolute and/or reference-dependent prognostic estimates. The primary outcome was prognostic accuracy relative to the LCPI.

**Results:**

Among 51 clinicians with complete responses, the median years in practice was 7 (interquartile range 3.5–19), 14 (27.5%) were female, 23 (45.1%) practiced in a community oncology setting, and baseline accuracy was 54.9% (95% confidence interval [CI] 47.0–62.8) across all vignettes. ML presentation improved accuracy (mean change relative to baseline 20.9%, 95% CI 13.9–27.9, *P* < 0.001). ML outputs using an absolute presentation strategy alone (mean change 27.4%, 95% 16.8–38.1, *P* < 0.001) or with reference dependence (mean change 33.4%, 95% 23.9–42.8, *P* < 0.001) improved accuracy, but reference dependence alone did not (mean change 2.0% [95% CI −11.1 to 15.0], *P* = 0.77). ML presentation did not change the rates of recommending ACP nor PC referral (mean change 1.3% and 0.7%, respectively).

**Limitations:**

The singular use case of prognosis in mNSCLC, low initial response rate.

**Conclusions:**

ML-based assessments may improve prognostic accuracy but not result in changed decision making.

**Implications:**

ML prognostic algorithms prioritizing explainability and absolute prognoses may have greater impact on clinician decision making.

Trial Registration: CT.gov: NCT06463977

**Highlights:**

- While machine learning (ML) algorithms may accurately predict mortality, the impact of prognostic ML on clinicians’ prognostic accuracy and decision making and optimal presentation strategies for ML outputs are unclear.
- In this multicenter randomized survey study among vignettes of patients with advanced cancer, prognostic accuracy improved by 20.9% when clinicians reviewed vignettes with a hypothetical ML mortality risk prediction, with absolute risk presentation strategies resulting in greater accuracy gains than reference-dependent presentations alone.
- However, ML presentation did not change the rates of recommending advance care planning or palliative care referral (1.3% and 0.7%, respectively).
- ML-based prognostic assessments without explanations improve prognostic accuracy but do not change decisions around palliative care referral or advance care planning.

Machine learning (ML) algorithms can improve the prognosis of disease-specific and overall survival in areas ranging from oncology to heart failure.^1^ Such ML algorithms can model linear and nonlinear relationships between large numbers of longitudinal variables to generate accurate individual-level prognoses, improving clinical decision support and individual-level risk stratification.^2^ These models are often based on routinely collected electronic health record or registry datasets and improve upon traditional clinical prognostic tools. Such tools usually rely on limited numbers of variables to estimate population-level survival or the intuition of clinicians, who significantly overestimate the prognosis for up to 70% of their patients.^3^

A prominent use case of ML-based prognostic tools is to identify high-risk patients with serious illnesses such as cancer who may benefit from early advanced care planning (ACP) and/or palliative care (PC) referral.^4–7^ Despite ACP and PC consultation being evidence- and guideline-based practices, 40% of patients with advanced cancer do not receive ACP nor PC prior to dying.^8–10^ While lack of time and perceived patient discomfort are cited barriers, another recognized barrier is lack of prognostic accuracy among clinicians, leading to overoptimistic prognoses.^3,11^ Overoptimistic prognosis or prognostic unawareness may lead to delayed or missed ACP conversations and/or PC or hospice referrals.^12^ Despite the promise of ML-based prognostic models in clinical decision support, prospective deployments have had variable impact on clinical processes such as ACP and PC utilization.^13–15^ Explainability and presentation strategy may contribute. The importance of explainability in prognostic ML models is unclear: behavioral interventions that present clinicians with “black-box” ML model estimates have still had a positive impact on ACP and PC. However, mixed-methods studies show that the black-box nature of such predictive estimates may decrease clinician trust in such predictions, decreasing uptake.^13,16–19^ Presentation strategy is also an understudied factor.^20^ Prognostic risk could be presented as an absolute prediction, in terms of 1-y mortality risk or months of survival. While providing an objective estimate, such presentations rely on accurate calibration of a model and may not give a sense of which patients ought to be prioritized. Another option is presenting “reference-dependent” predictions, derived from Kahneman’s prospect theory, which poses that predictions or choices made in reference to another set of options or observations may sometimes lead to desired decisions compared with presenting objective estimates only.^21^ For example, ML reference-dependent output could be presented in terms of the risk of a patient relative to others in a clinician’s or practice’s patient panel.

We conducted a factorial experiment using 3 hypothetical vignettes involving patients with lung cancer. Each vignette was associated with a literature-based gold-standard prognosis to facilitate studying prognostic accuracy.^22^ The objective of this study was to assess the impact of reference-dependent versus absolute prognostic estimates from a black-box ML prognostic algorithm on prognostic accuracy and decision making around ACP and PC. We also sought to determine how influential the ML prediction was, compared with standard clinical factors, in clinicians’ decision making.

## Methods

### Study Design

**Figure 1:**
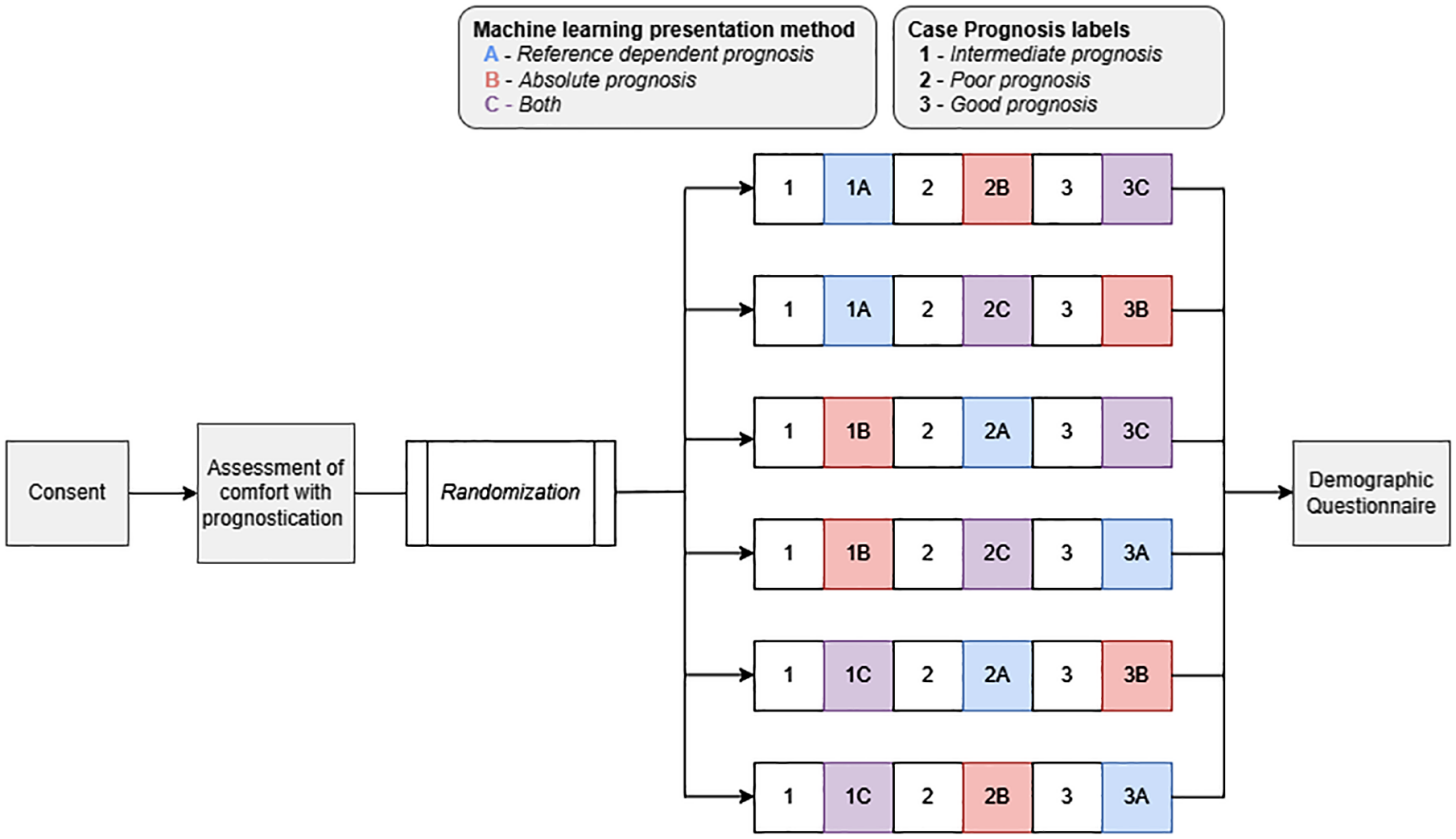
Trial schema. Schematic of randomization into case and presentation method. This was a 3 × 3 online factorial experiment employing a survey instrument hosted via Qualtrics presenting describing 3 patient vignettes. The study protocol is available in Supplement 1. Each vignette presented a patient with advanced non-small-cell lung cancer (aNSCLC). Factors that varied across the vignettes were 1) clinical prognosis (good, poor, or intermediate) and 2) risk prediction presentation strategies (reference dependent prognosis, absolute prognosis, or both). The vignettes included 2 parts: part 1 described a clinical case followed by questions on prognostic estimates and advance care planning and palliative care decisions, and part 2 presented the same clinical case plus a prognostic estimate from a hypothetical machine learning (ML) predictive algorithm. All respondents viewed clinical vignettes in the same order with respect to clinical prognosis. The order of presentation strategies for the ML risk predictions were randomized. This design led to 6 versions of the survey to which each participant was randomized.

The study consisted of a 3 × 3 online factorial experiment employing a survey instrument hosted via Qualtrics, a secure, HIPAA-compliant, Web-based survey application, describing 3 patient vignettes (Figure 1). The study protocol is available in Supplement 1. Each vignette presented a patient with advanced non-small-cell lung cancer (aNSCLC). Factors that varied across the vignettes were 1) clinical prognosis (good, poor, or intermediate) and 2) risk prediction presentation strategies (reference dependent prognosis, absolute prognosis, or both). The vignettes included 2 parts: part 1 described a clinical case followed by questions on prognostic estimates and ACP and PC decisions, and part 2 presented the same clinical case plus a prognostic estimate from a hypothetical ML predictive algorithm. All respondents viewed clinical vignettes in the same order with respect to clinical prognosis. The order of presentation strategies for the ML risk predictions were randomized. This design led to 6 versions of the survey to which each participant was randomized (Figure 1). This study was deemed exempt by the University of Pennsylvania Institutional Review Board #850382.

### Study Population and Recruitment

The study population consisted of a convenience sample of practicing medical oncologists (*N* = 70) who treated lung cancer in the United States, of whom 51 (73%) offered complete responses. A $40 virtual gift card was offered to anyone who completed the survey. Between March 13, 2023, and June 14, 2023, we recruited medical oncologists through direct emails to the principal investigator’s (R.B.P.) professional contacts (*n* = 27); direct messages via Doximity, an online networking service for medical professionals (*n* = 17); direct messages via X (formerly Twitter) (*n* = 6); and unspecified “other” (*n* = 1). Interested participants were directed to the study landing page, which confirmed study eligibility, provided additional study information, and provided informed consent prior to randomization. We used several methods to confirm that participants were medical oncologists. First, participants recruited via Doximity had a designation of “thoracic oncology” in their profile. Second, participants were recruited via X collated from existing thoracic oncology social media listservs. Third, all participants were required to self-report whether they were medical oncologists who treat lung cancer prior to proceeding to the consent section of the survey. Efforts were taken to sample equally from 4 US geographic regions (Northeast, South, Midwest, West) by dividing the original list of physicians into the 4 geographic regions (according to state listed in their social media or professional profile) and randomly sampling oncologists to approach within each list.

### Survey Development

#### Patient vignettes

We chose to present patients with aNSCLC because 1) a validated index, the Lung Cancer Prognostic Index (LCPI),^22^ could be used as a benchmark to define prognostic accuracy, and 2) early ACP and specialty PC referral—decisions assessed by the survey—have a strong evidence base in aNSCLC.^23–25^ We developed vignettes of patients with aNSCLC by varying clinical characteristics that are included in the LCPI. The 3 patient scenarios varied by clinical characteristics including age, gender, performance status, smoking history, extent of disease, symptoms, and molecular status (see Table 1). The 3 patient scenarios also differed by 1-y mortality risk, stratified by poor versus intermediate versus good prognosis, as benchmarked from the LCPI. Each of the 3 patient scenarios were presented in the same order for each subject: good prognosis first, poor prognosis second, and intermediate prognosis third.

**Table 1:** Vignettes with Prognostic Estimates and Life Expectancy

Clinical Scenario	Presentation Strategy for ML Algorithm Prediction^a^	Estimated Life Expectancy Based on a Prognostic Index^b^
Case 1: A 65-y-old woman who is a former 30 pack-year smoker presents with a cough, anorexia, and fatigue and is found to have a lung mass and several liver metastases with no other sites of metastatic disease. Endobronchial biopsy reveals a squamous cell carcinoma of the lung without an actionable mutation, PD-L1 15%. Her only major comorbidity is high blood pressure. She continues to work as a waitress, where she is on her feet for most of the day. Three months ago, she weighed 150 pounds. Today her weight is 140 pounds.	Absolute prognosis: A predictive algorithm identifies that her life expectancy is 18 to 24 mo.	21 mo
Case 2: A 60-y-old man who is a current smoker presents with hemoptysis and headaches. His weight has decreased from 210 to 160 pounds over the past 3 mo. Imaging reveals a lung mass and several brain metastases. He undergoes biopsy of 1 of the brain tumors, which reveals adenocarcinoma of lung origin without an actionable mutation, PD-L1 0%. He has moderate chronic obstructive pulmonary disease. He spends most of his day in a chair due to shortness of breath on exertion.	Reference-dependent prognosis: A predictive algorithm estimates that in comparison to your entire panel, he has a poor prognosis.	4.5 mo
Case 3: A 60-y-old woman who has never smoked is found to have a large right-sided lung mass and a solitary 2-cm brain metastasis. She exercises regularly and has no medical history. Computed tomography–guided biopsy shows an actionable epidermal growth factor receptor–mutated adenocarcinoma of the lung. She has no other sites of metastatic disease, and she has not lost any weight over the past 6 mo.	Both reference-dependent and absolute prognosis: A predictive algorithm estimates that her life expectancy is 30 to 40 mo and that in comparison to your entire panel, she has a good prognosis.	35 mo

ML, machine learning.

a Predictive information from the ML algorithm was presented only after clinicians had provided an initial prognostic estimate without the ML algorithm output.

b Life expectancy estimates were derived from median values from the Lung Cancer Prognostic Index, using variables contained in the vignettes. Median life expectancy was modified for some clinical vignettes based on feedback from a thoracic oncology focus group, prior to survey deployment.

#### Pilot testing

To confirm survey comprehension and face validity, trained research staff with expertise in mixed methods pilot tested the survey by completing a focus group (see Supplemental Methods) with 5 thoracic medical oncologists at a major research university hospital system. We received verbal consent from all participants to audio record the session, which was 60 min in duration and was professionally transcribed and analyzed for suggested changes. Each participant took the survey independently before engaging in a moderated discussion. This allowed us to address unexpected findings in preliminary data, adjust life expectancy estimates from the LCPI (heretofore termed “modified LCPI”) given potential changes in treatment paradigms, and make modifications to the survey to improve clarity and realism of the cases presented within. Specifically, our motivation to modify the median survival estimates from the LCPI were based on changes in prognosis for advanced lung cancer since the LCPI was trained. While we changed the median overall survival estimates based on our focus groups, we did not change the variables or overall risk classification (I–IV) that the LCPI outputs; thus, our designation of “good,” “intermediate,” and “poor” prognosis is still in line with the LCPI.

A summary of changes to the initial version of the survey is described in the Supplemental Methods. After making these changes, we readministered the survey to the focus group participants for a final round of feedback. We then made any necessary modifications to the survey prior to its national distribution. Each of the 5 participants in the focus group was offered $100 for participating.

#### Presentation of prognostic information

For each of the 3 vignettes, the presentation of prognostic information was operationalized in a pre–post manner. Part 1 involved case history without ML prediction, after which subjects responded to questions assessing various outcomes. Part 2 immediately followed and described the same vignette for the same patient with a single sentence that provided information from a hypothetical ML predictive algorithm.

There were 3 between-subjects presentation strategy conditions to present hypothetical mortality risk estimates: reference-dependent prognosis, absolute prognosis, and both (Table 1). In the reference-dependent strategy, prognosis was presented in relation to the subjects’ patient panel (e.g., “A predictive algorithm estimates that in comparison to your entire panel, she has an intermediate prognosis”). In the absolute strategy, prognosis was presented as a range centered around the modified LCPI estimate (e.g., an estimate of 21 mo was presented as “A predictive algorithm identifies that her life expectancy is 18 to 24 months.”). We chose to present absolute prognoses as a range rather than point estimate given general guidance from supportive care literature against presenting prognoses as point estimates.^26^ In the strategy that combined both conditions, prognosis was presented as first a range centered around the estimate, followed by a comparison to the physician’s panel (e.g., “A predictive algorithm estimates that her life expectancy is 18–24 months and that in comparison to your entire panel, she has an intermediate prognosis.“). The order of the vignettes in each survey was electronically randomized using simple randomization with regard to presentation strategies for ML risk predictions, creating 6 versions of the survey (Figure 1). Each participant was randomized to a survey version via the Qualtrics Randomizer function that was developed by the study team (W.J.F. and J.W.).

### Outcomes

The primary study outcome was prognostic accuracy. Secondary outcomes included decision making on 2 decisions influenced by prognostic estimates: advance care planning (ACP) and specialty PC referral (PC). Exploratory outcomes included clinician-reported contributors to prognosis.

#### Prognostic accuracy

Prognostic estimates were assessed after each of the 3 vignettes and measured by asking for anticipated life expectancy for the patient, in months. Prognosis outcomes were dichotomized as “accurate” versus “not accurate,” defined as in previous studies as whether the reported life expectancy estimate was within 33% of the modified LCPI estimate.^3^

#### Decision making

Decision making was assessed using 2 items administered after each of the 3 vignettes. The decisions assessed were 1) referral to PC at this point in the disease course and 2) ACP discussion at this point in the disease course. Each question was operationalized as a yes/no answer and was followed by a free response box asking, “Please share your reason for this decision.”

#### Contributors to prognosis

After each assessment of prognosis, participants were asked to assess which one of the following factors was the major contributor to their prognosis: age, smoking status, performance status, comorbidities, mutation status, symptoms, metastatic burden, or other. An additional option, “predictive algorithm,” was added after presentation of the ML prognostic estimate.

#### Covariates and moderators

Prior to the case vignettes, subjects were asked to rate their comfort level with estimating life expectancy and referring to PC. We measured comfort estimating life expectancy given known variability in clinicians’ extent and awareness of prognostic accuracy.^27^ After completion of the survey, several participant covariates that may influence rates of ACP or PC were assessed, including practice (geographic location, urbanicity, practice setting) and physician (years in practice, percentage of panel with lung cancer, gender identity, race, ethnicity, panel size) factors.^28,29^

### Statistical Analysis

The primary preplanned analysis compared prognostic accuracy in each of the 3 vignettes with or without ML presentation. This analysis specifically determined whether 1) any ML presentation improved mean prognostic accuracy, 2) any ML presentation reduced variation in prognoses (precision), and 3) whether presentation strategy affected prognostic accuracy.

Completed assessments in which physicians answered all 6 questionnaires were included in the analyses. Mean prognostic accuracy across vignettes was compared using a generalized estimating equation model with an exchangeable correlation structure,^30^ in which individual prognostic assessments were clustered within study participants to account for within-participant accuracy differences. Predictive margins and contrasts were calculated to estimate accuracy for each vignette, with 95% confidence intervals (CIs) calculated using the delta method.^31,32^ We compared variation in prognoses between presentation strategies using *F* tests. Two sensitivity analyses were performed. The first was an analysis of prognostic accuracy adjusted for respondent self-reported characteristics including dwelling (rurality), years in practice, whether the respondent primarily sees thoracic malignancies, gender, primary practice setting, size of patient panel, and comfort estimating life expectancy. The second was an analysis of prognostic accuracy using continuous prognostic accuracy, rather than a dichotomized accuracy measure. We defined the continuous prognostic accuracy variable as the absolute difference, in months, between the LCPI estimate used to generate our “accurate range” and the response provided in the questionnaire.

The secondary decision-making outcomes of PC and ACP were compared between the baseline versus ML presentations using McNemar’s test for paired responses before and after the ML presentation for each vignette.

We aimed to recruit 50 participants with 150 survey responses across all 3 vignettes. This ensured adequate power to detect a 10-percentage-point increase in prognostic accuracy in the ML versus no ML comparison. Comparisons between presentation strategies were regarded as exploratory. Statistical analyses were performed using Stata version 16 (StataCorp). Statistical significance was based on a *P* value <0.05. *P* values for multiple comparisons were not adjusted for in the preplanned analyses.

## Results

### Baseline Characteristics

Seventy patients responded to the survey and were randomized. Among 70 individuals randomized to a version of the survey, 51 (72.8%) offered complete responses to all questions after each vignette and were included in the primary analysis (Table 2). Study participants with complete responses were from 20 US states and the District of Columbia. Overall, median years in practice was 7 (interquartile range [IQR] 3.5–19), 14 (27.5%) were female, 23 (45.1%) practiced in a community oncology setting, and 20 (39.2%) saw primarily thoracic malignancies. Seven (13.7%) reported feeling uncomfortable estimating life expectancy, and no clinicians reported feeling uncomfortable with PC referral or ACP.

**Table 2:** Demographic Characteristics of Respondents

Variable	*n* (%)
*N*	51
Age, y, *n* (%)	
30–39	13 (26)
40–49	10 (20)
50–59	5 (10)
60–69	5 (10)
70–79	1 (2)
NA	17 (33)
Dwelling, *n* (%)	
Rural	5 (10)
Suburban	20 (39)
Urban	26 (51)
Years in practice, median (IQR)	7 (3, 19)
Primarily seeing thoracic malignancies, *n* (%)	20 (39)
Self-reported female gender, *n* (%)	14 (28)
Self-reported race, *n* (%)	
Asian	14 (28)
Other/prefer not to answer	9 (18)
White	28 (55)
Self-reported ethnicity, *n* (%)	
Hispanic or Latino	5 (10)
Not Hispanic or Latino	42 (2)
Prefer not to answer	4 (8)
Primary practice setting, *n* (%)	
National Cancer Institute designated	17 (33)
Other academic center	11 (22)
Community/private oncology practice/other practice	23 (45)
Size of patient panel, *n* (%)	
<1,500	35 (69)
≥1,500	13 (26)
Prefer not to answer	3 (6)
Comfort estimating life expectancy, *n* (%)	
Comfortable or somewhat comfortable	36 (70)
Neutral	8 (16)
Uncomfortable or somewhat uncomfortable	7 (14)
Comfort referring to PC, *n* (%)	
Comfortable or somewhat comfortable	50 (98)
Neutral	1 (2)
Uncomfortable or somewhat uncomfortable	0 (0)
Comfort referring to ACP (%)	
Comfortable or somewhat comfortable	50 (98)
Neutral	1 (2)
Uncomfortable or somewhat uncomfortable	0 (0)

ACP, advance care planning; IQR, interquartile range; NA, not applicable; PC, palliative care.

### Primary Outcome: Prognostic Accuracy

For the life expectancy estimation in years, baseline prognostic accuracy without ML was 54.9% (95% CI 47.0–62.8) across all 3 vignettes (Table 3). Baseline accuracy was 57.6% (95% CI 45.5–69.7) for the poor-prognosis case, 57.6% (95% CI 44.4–70.8) for the intermediate-prognosis case, and 49.5% (95% CI 36.1–62.8) for the good-prognosis case. ML presentation significantly improved prognostic accuracy overall (mean change in prognostic accuracy relative to baseline 20.9%, 95% CI 13.9–27.9; *P* < 0.001) and in each specific case (poor prognosis 20.0% [95% CI 13.1–26.8], *P* < 0.001; intermediate prognosis 20.6% [95% CI 12.9–28.3%], *P* < 0.001; good prognosis 22.2% [95% 14.3–30.1], *P* < 0.001) (eFigure 1). Compared with baseline presentations without ML, ML presentation strategies using an absolute presentation strategy alone (mean change 27.4% [95% 16.8–38.1], *P* < 0.001) or with reference dependence (mean change 33.4% [95% 23.9–42.8], *P* < 0.001) improved prognostic accuracy. Compared to baseline, a reference-dependence presentation strategy alone did not improve prognostic accuracy (mean change 2.0% [95% CI −11.1 to 15.0], *P* = 0.77) (Table 3). In sensitivity analyses, results were consistent when adjusting for covariates and when using a continuous outcome alone (Supplemental Tables S1 and S2).

**Table 3:** Prognostic Accuracy across Vignettes for Life Expectancy in Years

	Prognostic Accuracy (95% CI)	Mean Difference in Percentage Points from Baseline (95% CI)	*P*
Overall			
Baseline (no ML)	54.9 (47.0–62.8)	N/A	N/A
With ML	75.8 (69.0–82.6)	20.9 (13.9–27.9)	<0.001
Reference dependent	56.8 (43.5–70.2)	2.0 (−11.1 to 15.0)	0.768
Absolute	82.3 (71.8–92.8)	27.4 (16.8–38.1)	<0.001
Both	88.2 (79.5–96.9)	33.4 (23.9–42.8)	<0.001
Poor prognosis			
Baseline (no ML)	57.6 (45.5, 69.7)	N/A	N/A
Reference dependent	59.5 (42.2–76.9)	1.9 (−10.9, 14.8)	0.767
Absolute	83.9 (72.8–95.1)	26.3 (15.4, 37.3)	<0.001
Both	89.4 (79.7–99.1)	31.8 (22.6, 41.0)	<0.001
Intermediate prognosis			
Baseline (no ML)	57.6 (44.4, 70.8)	N/A	N/A
Reference dependent	59.5 (43.6–75.5)	1.9 (−11.0, 14.9)	0.768
Absolute	83.9 (71.5–96.4)	26.3 (16.0, 36.7)	<0.001
Both	89.4 (81.4–97.4)	31.8 (19.6, 44.1)	<0.001
Good prognosis			
Baseline (no ML)	49.5 (36.1, 62.8)	N/A	N/A
Reference dependent	51.5 (34.4–68.5)	2.0 (−11.3, 15.4)	0.768
Absolute	79.0 (66.4–91.7)	29.6 (16.4, 42.7)	<0.001
Both	85.9 (74.6–97.2)	36.4 (24.4–48.5)	<0.001

CI, confidence interval; ML, machine learning.

Prognostic estimates were assessed after each of the 3 vignettes and measured by asking for anticipated life expectancy for the patient, in months. Prognosis outcomes were dichotomized as “accurate” versus “not accurate,” defined as in previous studies as whether the reported life expectancy estimate was within 33% of the modified Lung Cancer Prognostic Index estimate. The mean difference from baseline was estimated using model contrasts. Confidence intervals were derived using the delta method.

**Figure 2:**
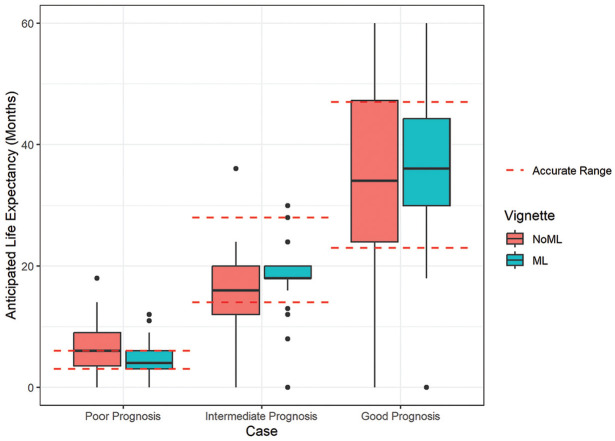
Red dotted lines delineate the range of prognostic accuracy, with outliers shown with black dots. Machine learning (ML) represents clinician responses aggregated across all 3 ML presentation strategies shown in eFigure1.

Compared with baseline responses without ML, providing ML estimates reduced variation in prognostic estimates for the poor-prognosis case (ratio of variances 2.17; *P* = 0.004) but not in the intermediate-prognosis (ratio of variances 1.54; *P* = 0.07) nor good-prognosis (ratio of variances 1.18; *P* = 0.30) cases (Figure 2). Among vignettes presented with ML prognoses, clinicians rated the ML estimate as the primary factor determining prognosis in 19.6% of cases (13.7% poor prognosis, 31.4% intermediate prognosis, 13.7% good prognosis) (Supplemental Table S3).

#### Secondary outcome: decision making

**Figure 3:**
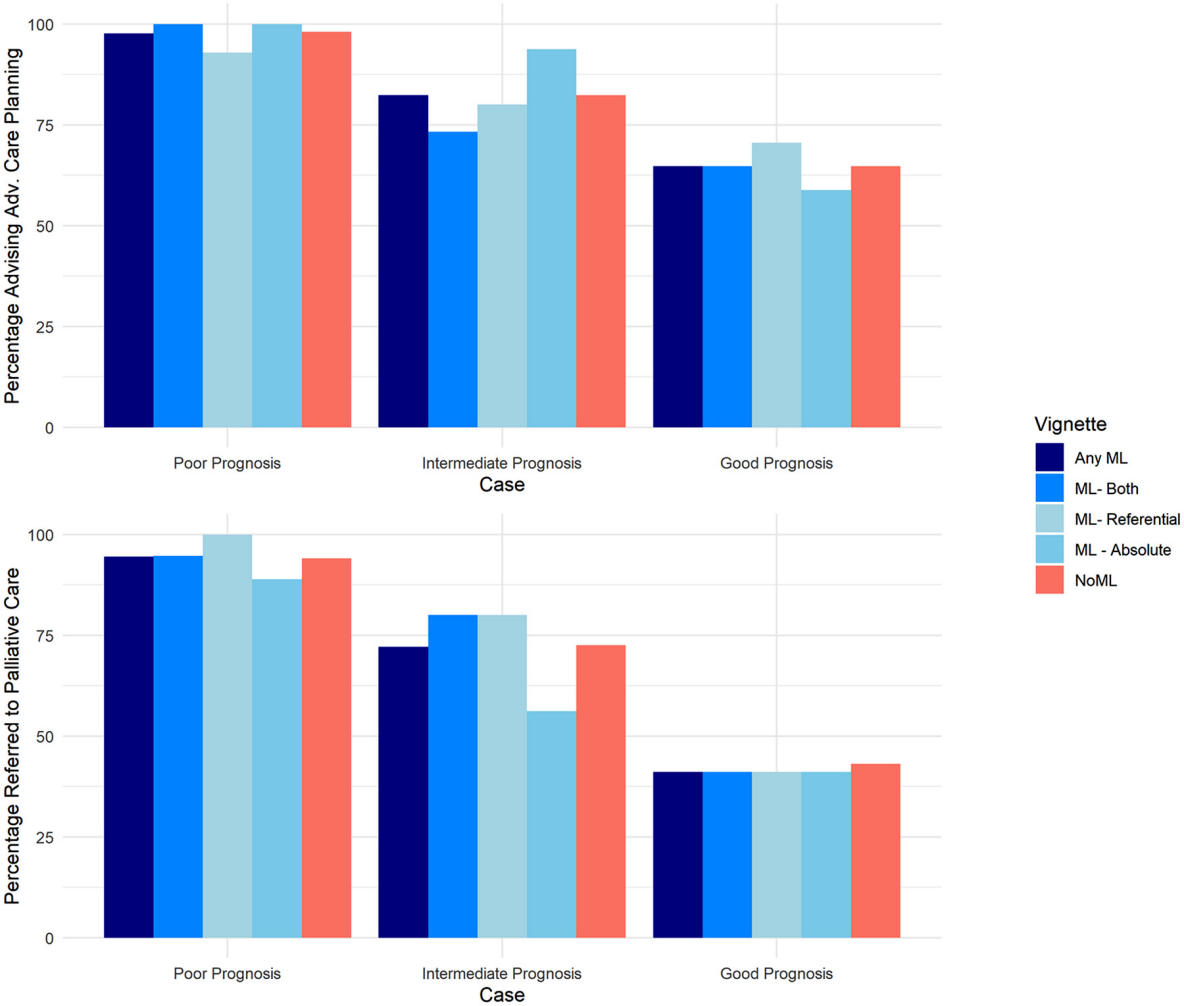
Decision making before and after the machine learning (ML) presentation. Clinicians were asked whether they would refer the patient to palliative care or advanced care planning. The percentage of clinicians who would refer to each service is shown before and after the ML presentation.

At baseline prior to any ML presentation, physicians recommended ACP in 81.7% of cases (98.0% poor prognosis, 82.4% intermediate prognosis, 64.7% good prognosis) and PC in 69.9% of cases (94.1% poor prognosis, 72.5% intermediate prognosis, 43.1% good prognosis). ML presentation did not change the rates of recommending ACP (1.3% of recommendations changed from baseline after ML presentation, *P* = 1.0) nor PC referral (0.7% of recommendations changed from baseline after ML presentation, *P* = 1.0) (Figure 3). These findings were consistent across prognostic risk groups and ML presentation strategies (Supplemental Tables S2 and S4). For participants who responded that patients did not need ACP or PC in the baseline setting, 1.3% and 0.6%, respectively, changed their answer after ML presentation.

## Discussion

In this randomized vignette-based study, ML-based prognostic assessments improved the accuracy of prognosis, with the presentation of ML outputs using absolute estimates resulting in greater accuracy gains than a reference-dependent strategy alone. Despite improving accuracy and, for poor-prognosis cases, reducing the variability of predictions, providing ML estimates did not change decision making around ACP and PC, 2 key decisions informed by more accurate prognosis.^26^ This informs expectations about deployment of advanced prognosis algorithms in routine workflows.

Previous experimental vignette-based studies investigated how ML-based models in imaging affect diagnostic accuracy. One study using vignettes involving chest imaging showed that clinician accuracy increased with accurate ML predictions, particularly for those providing explanations. Even when model estimates were systematically biased and inaccurate, explanations modestly improved accuracy.^19^ In another study among 140 radiologists across 15 diagnostic tasks around chest X-rays, diagnostic performance after AI outputs varied by clinicians and did not depend on clinician experience but was lower after inaccurate AI predictions.^33^ Related work suggests that in some radiology cases, radiologists overprioritize their own intuition above accurate AI—so called “own-information” bias.^34^

Our study extends these findings among diagnostic AI by studying the use case of a prognostic ML algorithm, which has been increasingly deployed in settings such as sepsis and mortality prediction. Furthermore, our study examines whether presentation strategy results in differential effects on accuracy and decision making. Our study presumed an accurate ML algorithm anchored to gold-standard literature-based estimates derived from prospective studies.^22^ While prospect behavioral economic theory suggests that reference-dependent predictions may sometimes lead to desired decisions, reference dependence can also lead to biases in decision making, as individuals may place undue weight on the reference point, even when it is not an accurate representation of the available options.^21^ Our study shows that this theory extends to the setting of medical prognosis, where ML algorithms led to improved prognostic accuracy only when anchored to absolute prognoses rather than reference-dependent presentations. Providing absolute prognostic estimates is controversial in fields such as oncology and PC, in which prognosis is uncertain and it is critical to move beyond numeric prognosis to explore patient goals and wishes. However, if algorithmic strategies are used for prognosis, our study suggests that absolute estimates are likely critical to ensure accuracy.

It is notable that despite improving prognostic accuracy, providing ML estimates did not improve decision making around ACP or PC, which is guideline based for all patient vignettes in this study. This stands in contrast to other clinical settings such as cancer screenings, in which similar vignette-based studies have shown risk algorithm estimates do influence decision making around referrals for screening.^35^ Rates of recommending PC or ACP in the baseline setting—without ML prediction—were higher than reported in practice. This may reflect a Hawthorne effect that differs from previous experimental evidence using actual patient cases, in which prognosis is routinely overestimated.^3^ However, even in settings where prognostic accuracy improved after ML presentation, there was very little impact on PC and ACP decisions. The minimal change in decision making after ML presentation suggests that, despite modifying prognoses after ML, physicians may anchor on their pre-ML decisions when making ACP/PC decisions post-ML. This may reflect some threshold-based decision making by clinicians, in that ML outputs did not change predicted risk sufficiently to exceed a threshold that would result in a change decision. This may also reflect self-perceived resource constraints in the time or staffing available for ACP or PC. Despite guidance that ACP and PC is appropriate in any stage IV disease, rates were notably lower for good-prognosis cases, suggesting that clinicians still triage ACP/PC based on prognosis. This is consistent with retrospective evidence suggesting lower numeric rates of PC referrals for advanced cancers with better prognosis (e.g., prostate, breast) than advanced cancers with poorer prognosis (e.g., pancreatic, lung).^36,37^ This confirmation bias presents a risk to the likely impact of ML algorithms in clinical care workflows. Furthermore, physicians rated ML predictions as having relatively low importance relative to other clinical factors in influencing decision making. This mirrors work suggesting that clinicians discount clinical guidelines relevant to other patient factors and their own experience.^38^ These findings together suggest that providing raw ML predictions without explanations are unlikely to meaningfully improve prognosis. It is also possible that, in contrast to influencing prognostication, influencing clinician decision making may require presenting evidence that algorithms are well validated. Explainability in the prediction setting is an emerging field but should be prioritized in the predictive analytic setting.^39^ Such efforts should be complemented with efforts to improve ML literacy.

### Limitations

There are several limitations to this analysis. First, we study the singular use case of prognosis in advanced lung cancer. This was intentional, given the presence of a validated prognostic index to enable comparisons of prognostic accuracy across vignettes of various risk to a gold standard. While patterns of responses to lung cancer cases may differ from other diseases such as heart and liver failure, guidelines around referral to PC and ACP are less well engrained for these conditions. In contrast, referral to specialty PC and ACP in advanced lung cancer is an evidence-based practice, and thus, we could study the impact of ML in relation to a standard of 100%. Second, our definition of prognostic accuracy was intentionally lenient, classifying a range of values around a gold standard as accurate. While definitions of accuracy may differ, this strategy has been similarly used in other vignette-based studies of prognostic accuracy.^3^ Responses classified as “inaccurate” differed by median life expectancy by more than 12 mo in some cases, which may have large implications on decisions around end-of-life chemotherapy and other treatments.^40^ Third, our survey was limited by the lower response rate to email and social media outreach, preventing more extensive subgroup analyses. However, as illustrated by Table 1, we were still able to capture diverse representation across certain demographic characteristics, practice setting and location, and comfort level with prognosis. Furthermore, our analysis compared participants’ responses before and after the ML prediction, accounting for known physician-level differences in prognostic accuracy and the propensity to refer to PC.

## Conclusions

In this randomized vignette-based study, ML-based prognostic assessments based on a literature-based index improved the accuracy of clinicians’ prognosis but did not result in changes in key decisions around PC referral and ACP. Presentation strategies using absolute predictions resulted in greater improvements in accuracy than reference-dependent predictions alone. This study informs expectations for ML prognostic decision aids on real-world decision making and argues for careful presentation and explainability in AI-based prognostic decision support. Future directions of this work include adding components of explainability to vignettes to explore the impact of greater algorithmic transparency on prognosis and decision making. In addition, these experiments should be applied to other clinical contexts in which AI is growing in prominence, including large language model–based history summarization and radiology-based AI decision support for diagnosis.

## Supplemental Material

sj-docx-1-mdm-10.1177_0272989X251349489 – Supplemental material for The Impact of Machine Learning Mortality Risk Prediction on Clinician Prognostic Accuracy and Decision Support: A Randomized Vignette Study Supplemental material, sj-docx-1-mdm-10.1177_0272989X251349489 for The Impact of Machine Learning Mortality Risk Prediction on Clinician Prognostic Accuracy and Decision Support: A Randomized Vignette Study by Ravi B. Parikh, William J. Ferrell, Anthony Girard, Jenna White, Sophia Fang, Justin E. Bekelman and Marilyn M. Schapira in Medical Decision Making
