# Supplementary material for: The Impact of Machine Learning Mortality Risk Prediction on Clinician Prognostic Accuracy and Decision Support: A Randomized Vignette Study
Source: Med Decis Making. 2025 Jul 4;45(6):690–702. doi: 10.1177/0272989X251349489 (PMC12233153; doi:10.1177/0272989X251349489)
Supplement: sj-docx-1-mdm-10.1177_0272989X251349489 – Supplemental material for The Impact of Machine Learning Mortality Risk Prediction on Clinician Prognostic Accuracy and Decision Support: A Randomized Vignette Study [file sj-docx-1-mdm-10.1177_0272989X251349489.docx]

**Multimedia Appendix Material**

Title: The Impact of Machine Learning Mortality Risk Prediction on Clinician Prognostic Accuracy and Decision Support: A randomized vignette study

Study authors: Ravi B. Parikh, MD, MPP, William J. Ferrell, MPH, Anthony Girard, ScM, Jenna White, MSW, Sophia Fang, Justin E. Bekelman, MD, Marilyn M. Schapira, MD, MPH

**Table of Contents**

Supplemental Methods

- Twitter Recruitment Language
- Summary of changes based on focus group feedback

Supplemental Figure 1. Prognostic accuracy by case type

Supplemental Table 1. Prognostic accuracy across vignettes for life expectancy in years, adjusted for respondent self-reported characteristics

Supplemental Table 2. Prognostic accuracy across vignettes for life expectancy in years (Original continuous prognostic accuracy)

Supplemental Table 3. Top listed reasons for prognostic assessments

Supplemental Table 4. Decision-making before and after ML presentation

**Supplemental Methods**

**Twitter Recruitment Language**

*Calling all Medical Oncologists who treat lung cancer— help us learn about prognostic decision aids as part of a NIH-funded study. Complete this 15 min survey - https://bit.ly/3KA5RbQ. We offer an incentive as a thank you. #LCSM #MedTwitter #OncTwitter #ASCO Please RT!*

**Summary of changes based on focus group feedback:**

Based on the focus group’s feedback, we made changes to the survey’s overall presentation, its questions’ clarity and comprehensibility, and individual cases’ plausibility and prognostic accuracy. With regards to the survey’s presentation, we better emphasized that the second version of each case was the same case as before through language and visual cues (e.g., font weight; including a preface that read, “consider the same patient again summarized below”). We also made visual changes such as changing the background color from blue to white for legibility and altering paragraph spacing to give greater clarity on which sentences were connected. Additionally, we changed the framing of some questions. This included specifying that respondents should select the factor that most influenced their estimated prognostic rather than asking for the reason, which assumes there is only one, and adding a preface that the research team was specifically looking at whether the additional information of a machine learning estimate affected their estimates. We also moved from asking about referral to “palliative care” to referral to “a palliative care specialist” due to acknowledgement of the differences of what “palliative care” looks like across settings. Further, we changed timing of their potential referral from “now,” to “at this point in their disease course,” as participants brought up confusion over when “now” was and concerns about immediately referring any new patient to palliative care. Lastly, we changed several aspects of the cases themselves. This included altering patient age or weight to better align with the assigned prognosis, or the prognosis to fit participants’ estimates. We also added more details, such as describing a patient as a “former 30-pack-year smoker” rather than as a “former smoker” as one participant expressed that they presumed how significant the patient’s smoking was, and including a reason for why one patient was mostly sedentary, as another stated their intent to treat would have to take into account whether this was due to cancer or a comorbidity.

Supplemental Figure 1. Prognostic accuracy by case type


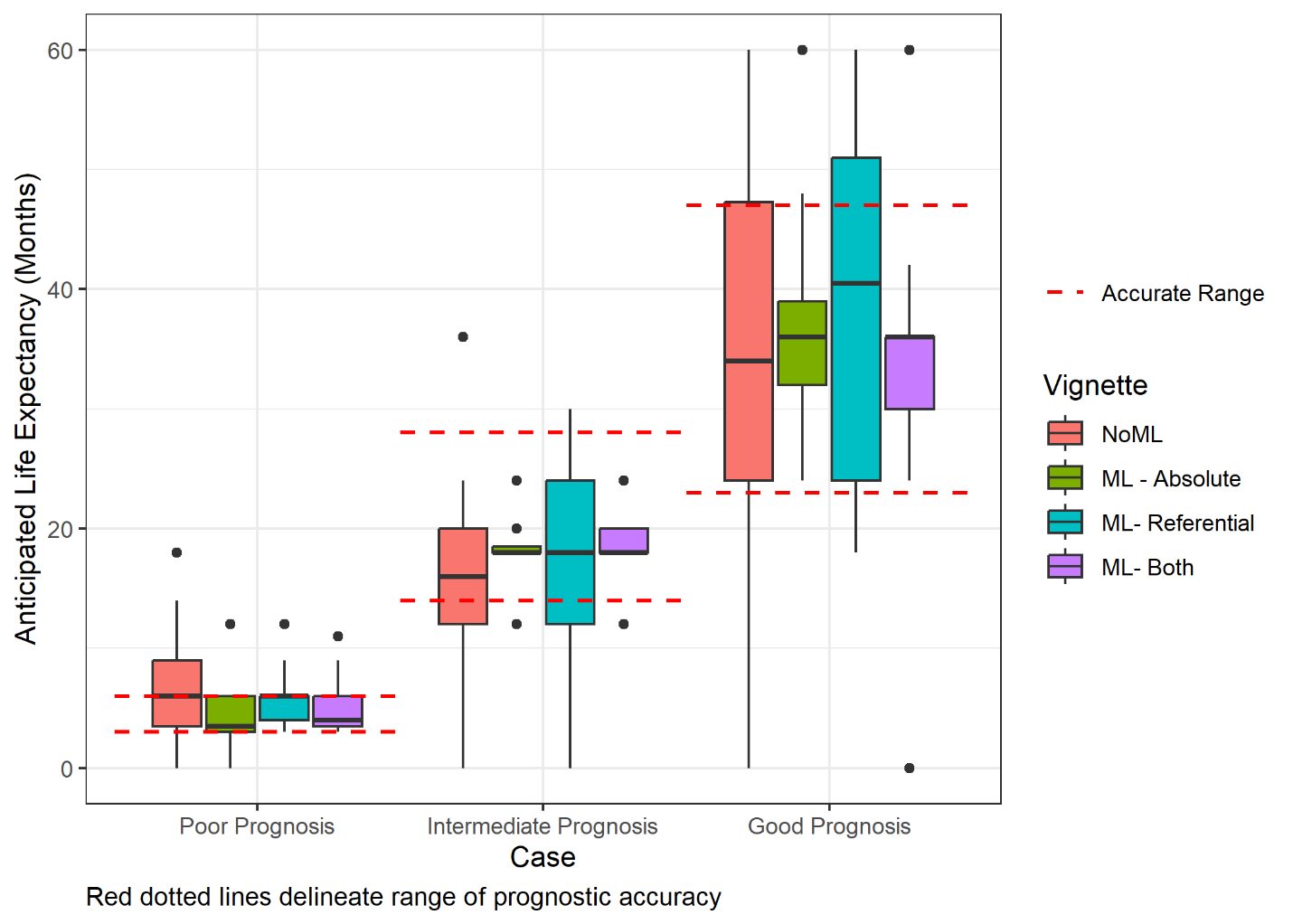


| **Supplemental Table 1. Prognostic accuracy across vignettes for life expectancy in years, adjusted for respondent self-reported characteristics** | | | |
| --- | --- | --- | --- |
|  | Prognostic accuracy (95% CI) | Mean difference in percentage-points from baseline (95% CI) | p |
| Overall |  |  |  |
| Baseline (No ML) | 61.5 (45.9, 77.1) | N/A | N/A |
| With ML | 81.3 (71.2, 91.5) | 19.8 (10.8, 28.8) | <.001 |
| Reference-dependent | 63.6 (41.8, 85.4) | 2.1 (-12.2, 16.4) | .774 |
| Absolute | 88.0 (78.2, 97.7) | 26.4 (13.9, 38.9) | <.001 |
| Both | 92.4 (86.0, 98.9) | 30.9 (17.4, 44.4) | <.001 |
| Poor-prognosis |  |  |  |
| Baseline (No ML) | 64.6 (47.5, 81.7) | N/A | N/A |
| Reference-dependent | 66.6 (43.2, 90.1) | 2.0 (-11.7, 15.8) | .773 |
| Absolute | 89.4 (79.9, 98.8) | 24.7 (11.8, 37.7) | <.001 |
| Both | 93.4 (86.6, 100) | 28.8 (15.2, 42.3) | <.001 |
| Intermediate-prognosis |  |  |  |
| Baseline (No ML) | 64.3 (44.1, 84.6) | N/A | N/A |
| Reference-dependent | 66.4 (42.2, 90.6) | 2.0 (-11.9, 15.9) | .774 |
| Absolute | 89.3 (78.0, 100) | 24.9 (11.1, 38.7) | <.001 |
| Both | 93.3 (87.1, 99.5) | 29.0 (11.8, 46.1) | <.001 |
| Good-prognosis |  |  |  |
| Baseline (No ML) | 55.6 (35.6, 75.7) | N/A | N/A |
| Reference-dependent | 57.8 (32.3, 83.4) | 2.2 (-13.0, 17.4) | .776 |
| Absolute | 85.2 (73.1, 97.3) | 29.6 (14.3, 44.9) | <.001 |
| Both | 90.6 (81.9, 99.4) | 35.0 (18.9, 51.1) | <.001 |
| CAPTION: Prognostic estimates were assessed after each of the 3 vignettes and measured by asking for anticipated life expectancy for the patient, in months. Prognosis outcomes were dichotomized as “accurate” vs. “not accurate”, defined as in previous studies as whether the reported life expectancy estimate was within 33% of the modified LCPI estimate. Mean difference from baseline was estimated using model contrasts. Confidence intervals were derived using the delta method. | | | |

|  | | | |
| --- | --- | --- | --- |
| **Supplemental Table 2. Prognostic accuracy across vignettes for life expectancy in years (Original continuous prognostic accuracy)** | | | |
|  | Prognostic accuracy, months (95% CI) | Mean difference in months from baseline (95% CI) | p |
| Overall |  |  |  |
| Baseline (No ML) | 7.9 (6.5, 9.2) | N/A | N/A |
| With ML | 6.0 (4.6, 7.3) | -1.9 (-2.6, -1.2) | <.001 |
| Reference-dependent | 9.3 (6.2, 12.4) | 1.4 (-0.8, 3.7) | .210 |
| Absolute | 4.4 (3.0, 5.8) | -3.5 (-5.1, -1.8) | <.001 |
| Both | 4.1 (2.4, 5.8) | -3.7 (-5.4, -2.1) | <.001 |
| Poor-prognosis |  |  |  |
| Baseline (No ML) | 3.6 (3.0, 4.2) | N/A | N/A |
| Reference-dependent | 5.1 (2.7, 7.4) | 1.4 (-0.8, 3.7) | .210 |
| Absolute | 0.2 (-1.3, 1.7) | -3.5 (-5.1, -1.8) | <.001 |
| Both | -0.1 (-1.8, 1.6) | -3.7 (-5.4, -2.1) | <.001 |
| Intermediate-prognosis |  |  |  |
| Baseline (No ML) | 6.4 (5.3, 7.6) | N/A | N/A |
| Reference-dependent | 7.9 (5.5, 10.2) | 1.4 (-0.8, 3.7) | .210 |
| Absolute | 3.0 (1.2, 4.7) | -3.5 (-5.1, -1.8) | <.001 |
| Both | 2.7 (0.8, 4.6) | -3.7 (-5.4, -2.1) | <.001 |
| Good-prognosis |  |  |  |
| Baseline (No ML) | 13.5 (10.1, 17.0) | N/A | N/A |
| Reference-dependent | 15.0 (9.9, 20.1) | 1.4 (-0.8, 3.7) | .210 |
| Absolute | 10.1 (7.0, 13.1) | -3.5 (-5.1, -1.8) | <.001 |
| Both | 9.8 (6.5, 13.1) | -3.7 (-5.4, -2.1) | <.001 |
| CAPTION: Prognostic estimates were assessed after each of the 3 vignettes and measured by asking for anticipated life expectancy for the patient, in months. Prognosis outcomes were dichotomized as “accurate” vs. “not accurate”, defined as in previous studies as whether the reported life expectancy estimate was within 33% of the modified LCPI estimate. Mean difference from baseline was estimated using model contrasts. Confidence intervals were derived using the delta method. | | | |

| **Supplemental Table 3. Top listed reasons for prognostic assessments** | | |
| --- | --- | --- |
|  | Baseline (no ML) | Machine learning |
| Poor-prognosis case, n (%) |  |  |
|  | Performance Status, 22 (43.1%) | Performance Status, 25 (49.0%) |
|  | Metastatic Burden, 17 (33.3%) | Metastatic Burden, 12 (23.5%) |
|  | Symptoms, 7 (13.7%) | Predictive Algorithm, 7 (13.7%) |
| Intermediate-prognosis case, n (%) |  |  |
|  | Performance Status, 25 (49.0%) | Performance Status, 16 (31.4%) |
|  | Metastatic Burden , 14 (27.5%) | Predictive Algorithm, 16 (31.4%) |
|  | Mutation Status, 9 (17.6%) | Metastatic Burden, 9 (17.6%) |
| Good-prognosis case, n (%) |  |  |
|  | Mutation Status, 39 (76.5%) | Mutation Status, 35 (68.6%) |
|  | Metastatic burden, 6 (11.8%) | Predictive Algorithm, 7 (13.7%) |
|  | Performance status, 4 (7.8%) | Metastatic Burden, 4 (7.8%) |

NOTE: In the baseline survey, predictive algorithm was not an option to choose.

| **Supplemental Table 4. Decision-making before and after ML presentation** | | | | | | | | | |
| --- | --- | --- | --- | --- | --- | --- | --- | --- | --- |
|  |  | Any ML prognostic estimate (n=153) | | Reference-dependent presentation (n=51) | | Absolute presentation strategy (n=51) | | Combined strategy (n=51) | |
|  |  | Recommend ACP (%) | Did not recommend ACP (%) | Recommend ACP (%) | Did not recommend ACP (%) | Recommend ACP (%) | Did not recommend ACP (%) | Recommend ACP (%) | Did not recommend ACP (%) |
| Baseline (Prior to ML presentation) (n=153) | Recommended ACP | 124 (99.2%) | 1 (0.8%) | 41 (97.7%) | 1 (2.3%) | 43 (100%) | 0 (0%) | 40 (100%) | 0 (0%) |
|  | Did not recommend ACP | 1 (3.6%) | 27 (96.4%) | 0 (0%) | 9 (100%) | 0 (0%) | 8 (100%) | 1 (9.1%) | 10 (90.9%) |
|  |  |  |  |  |  |  |  |  |  |
|  |  | Any ML prognostic estimate (n=153) | | Reference-dependent presentation (n=51) | | Absolute presentation strategy (n=51) | | Combined strategy (n=51) | |
|  |  | Recommend PC (%) | Did not recommend PC (%) | Recommend PC (%) | Did not recommend PC (%) | Recommend PC (%) | Did not recommend PC (%) | Recommend PC (%) | Did not recommend PC (%) |
| Baseline (Prior to ML presentation) (n=153) | Recommended PC | 106 (99.1%) | 1 (0.9%) | 37 (100%) | 0 (0%) | 32 (97.0%) | 1 (3.0%) | 37 (100%) | 0 (0%) |
|  | Did not recommend PC | 0 (0%) | 46 (100%) | 0 (0%) | 14 (100%) | 0 (0%) | 18 (100%) | 0 (0%) | 14 (100%) |
